# Notes Relative to the Buffalo Lithia Water

**Published:** 1892-04

**Authors:** William A. Hammond

**Affiliations:** Washington, D. C.


### NOTE RELATIVE TO THE BUFFALO LITHIA WATER.

BY WILLIAM A. HAMMOND, M. D.

There is a point in relation to the therapeutical efficacy of the Buffalo Lithia Water which has not yet, I think, received sufficient attention. It is well known that many cases of diseases of the nervous system are complicated with lithæmia, and that unless this condition is removed, a cure is very often retarded and not infrequently entirely prevented. It is quite commonly the case that in cerebral congestion producing insomnia, nervous prostration resulting from over-mental work, or much emotional disturbance, and in epilepsy (to say nothing of many cases of insanity,) an excess of uric acid in the blood is often observed. This state appears to be altogether independent of the character of the food, for no matter how careful the physician may be in regard to the diet of his patient the lithæmic condition continues. I have tried to overcome this persistence by the use of phosphate of ammonia and other so-called solvents for uric acid, but without notable effect.

Several years ago, however, I began to treat such cases with Buffalo Lithia Water with a result that was as astonishing to me as it was beneficial to the patient, so that now in all cases of nervous diseases under my charge, in which there is an excess of uric acid in the blood, I use Buffalo Lithia Water in large quantities. By this, I mean that I do not have the patient drink merely a tumbler or two in the course of the day, but that I flood him, so to speak, with the water, making him drink a gallon or even more, in the twenty-four hours. By this course the urine after a few days ceases to deposit uric acid crystals on standing, the morbid irritability of the patient disappears, the tongue becomes clean, the wandering pains in the head are abolished, and the system is rendered much more amenable to the special treatment which may be necessary for the cure of the disease from which the patient suffers.

I have tried carbonate of lithia dissolved in water in various proportions, but it certainly does not, in cases to which I refer, have the same effect as Buffalo Lithia Water.

Washington, D. C., Jan. 25, 1892.

The committee appointed at the last meeting of the American Medical Association to consider the best means for promoting the prosperity of the sections of the association, will hold an adjourned meeting at the Hotel Cadillæ, Detroit, Mich., June 6, at 3 P. M.

Members of the committee are requested to notify the chairman of their intention to be present at this meeting.

The committee would esteem it a favor if each member of the association would communicate in writing his or her views concerning the best measures for promoting the development of the sections. Such communications may be sent to the chairman of the committee.

JOHN S. MARSHALL, M. D., Chairman.
9 Jackson St., Chicago.

---

Dr. C. S. Robinson, Richford, Tiago Co., N. Y., says: I have tried papine (Battle & Co.) and I find it possesses the medicinal virtues of opium, unalloyed with the drawbacks following the use of other forms of the drug. I tested papine in my own case, having used many forms of opium, during forty years, but only in acute attacks. It is not harmful like crude opium, morphine and other preparations, in delicate or irritable stomachs; on the contrary, it is acceptable as cordial. Also, the head is not made ill as it is by the other forms of opium that have come under my observation during most half a century. Papine is more prompt than morphine, except when the latter is used hypodermically. My wife has acute rheumatic attacks, and so-called "sick-headaches," and long ago decided she was unable to bear morphine or opium treatment. On hearing me extol papine, she tried it unbeknown to me, and afterwards reported, saying: "I believe it is indeed a good remedy; I can take it, for it does not make me sicker when I am sick."

